# Coherent and Tunable Terahertz Radiation from Graphene Surface Plasmon Polarirons Excited by Cyclotron Electron Beam

**DOI:** 10.1038/srep16059

**Published:** 2015-11-03

**Authors:** Tao Zhao, Sen Gong, Min Hu, Renbin Zhong, Diwei Liu, Xiaoxing Chen, Ping Zhang, Xinran Wang, Chao Zhang, Peiheng Wu, Shenggang Liu

**Affiliations:** 1 Terahertz Research Center, School of Physical Electronics, University of Electronic Science and Technology of China, Chengdu, Sichuan, 610054, China,; 2 Cooperative Innovation Centre of Terahertz Science, Chengdu, Sichuan, 610054, China; 3 School of Electronic Science and Engineering, Nanjing University, Nanjing, Jiangsu, 210000, China; 4 School of Physics and Institute for Superconducting and Electronic Materials, University of Wollongong, New South Wales 2522, Australia

## Abstract

Terahertz (THz) radiation can revolutionize modern science and technology. To this date, it remains big challenges to develop intense, coherent and tunable THz radiation sources that can cover the whole THz frequency region either by means of only electronics (both vacuum electronics and semiconductor electronics) or of only photonics (lasers, for example, quantum cascade laser). Here we present a mechanism which can overcome these difficulties in THz radiation generation. Due to the natural periodicity of 2π of both the circular cylindrical graphene structure and cyclotron electron beam (CEB), the surface plasmon polaritions (SPPs) dispersion can cross the light line of dielectric, making transformation of SPPs into radiation immediately possible. The dual natural periodicity also brings significant excellences to the excitation and the transformation. The fundamental and hybrid SPPs modes can be excited and transformed into radiation. The excited SPPs propagate along the cyclotron trajectory together with the beam and gain energy from the beam continuously. The radiation density is enhanced over 300 times, up to 10^5^ W/cm^2^. The radiation frequency can be widely tuned by adjusting the beam energy or chemical potential. This mechanism opens a way for developing desired THz radiation sources to cover the whole THz frequency regime.

Over the past decades, terahertz (THz) radiation becomes one of the most intensive research fields in modern science and technology because of its unique characteristics and wide potential applications^1^,^2^,^3^,^4^. It remains a great challenge to develop coherent and tunable THz radiation sources with high power density. The electronic devices struggle to generate waves much above 500 GHz^5^,^6^,^7^. And infrared sources become very dim as the frequency approaches the THz region^6^. From the photonics side, further reducing the frequency and increasing the operating temperature with the QCL technology is extremely challenging^6^,^8^,^9^. Therefore, up to now, development of desired THz radiation sources to meet the requirements for the rapidly growing applications is still an outstanding challenge.

Recently advancement in graphene research opened up new opportunities to develop THz sources due to graphene’s unique and superior electronic and optical properties^10^,^11^,^12^. Many of graphene’s potential applications^13^,^14^,^15^,^16^,^17^,^18^ have been demonstrated. However, its use in THz radiation sources is still lacking. Theoretical and experimental studies demonstrated that graphene sheet supports surface plasmon polaritons (SPPs) with frequencies in THz and mid-infrared regimes^19^,^20^. Graphene SPPs exhibit remarkable features such as stronger mode confinement and lower propagation loss compared to those in noble metal films^21^,^22^,^23^. More importantly, the properties of graphene SPPs can be tuned by adjusting electrostatic gating or chemical doping^14^,^15^,^24^. Based on the electrically controllable feature of SPPs, a unidirectional SPPs launcher with quite high extinction ratio and generation efficiency was demonstrated^25^. And in nanostructured graphene, such as nanodisks, nanorings, and nanoribbons, up to 30% high absorption efficiency and several micrometers operating wavelength were achieved through directly coupling between SPPs and incident light^26^,^27^,^28^.

It is known that SPPs in planar graphene always lie below the light line in dielectrics and thus cannot be transformed into radiation. This momentum mismatch can be removed if a periodic dielectric substrate is used^17^,^18^. Fortunately, the circular cylindrical structure and CEB both have a natural periodicity of 2π. In what follows we shall show that SPPs modes can indeed cross the light line of the dielectrics and transformation of SPPs into radiation becomes possible. It has been shown that circular cylindrical graphene structure can keep the main electronic and optical properties of those of planar graphene sheet provided when the radius is large enough^29^,^30^,^31^,^32^. We shall demonstrate that the circular cylindrical graphene structure is an excellent candidate for THz sources which are coherent, with high power density and great tunability. Our results shall significantly broaden applications of graphene-based plasmonics in science and technology^15^,^33^,^34^.

Based on the natural periodicity of both the structure and CEB, we present a novel physical mechanism of generation of THz radiation. The mechanism involves two processes, (i) excitation of SPPs in circular cylindrical graphene structures with a CEB inside the light cone of the dielectrics, and (ii) immediate transformation of the excited SPPs into Cherenkov THz radiation. Our theoretical analysis and numerical simulation show that, based on this mechanism, the room temperature, coherent, tunable THz radiation sources with high power density, can be developed. Moreover, the SPPs are propagating along the cyclotron trajectory together with the electron beam, maintaining synchronization between SPPs and CEB in both the angular velocity and longitudinal phase-velocity. This synchronization assures that SPPs can gain energy from the electron beam continuously to compensate the loss due to the radiation and decay. Both monolayer and double-layer graphene structures are proposed and studied. In case of the double-layer structure, two-color THz radiation can be achieved.

## Results

### Dispersion equation of the circular cylindrical monolayer graphene structure

**Figure 1:**
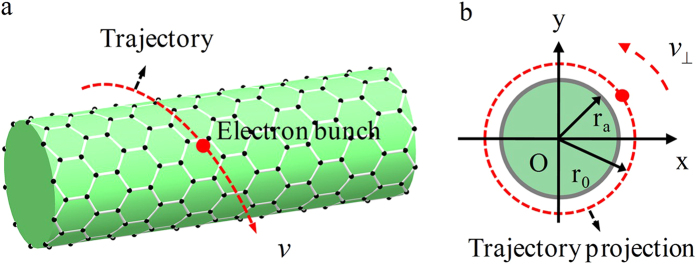
Schematic of the circular cylindrical monolayer graphene structure with dielectric loading. Three-dimensional view (**a**) and side view (**b**) of the structure. The radius of the dielectric medium is r_a_, the radius of the trajectory projection of CEB in X-Y plane is r_0_, it moves at a velocity 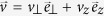 above the graphene layer with a cyclotron trajectory, 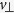 is the cyclotron velocity of the beam around z axis, and *v*_*z*_ is the z component of velocity.

The circular cylindrical monolayer graphene structure with dielectric loading is shown in Fig. 1, CEB is moving along r = r_0_ with velocity 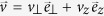. A monolayer graphene is coated on the dielectric medium. Graphene is considered to be infinitely thin with conductivity 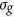
29,30,31,32.

The detailed mathematical manipulations of electromagnetic fields produced by the CEB, dispersion relation and output power density for the structure can be found in Appendix I and II of the Supplementary materials. The dispersion equation is,


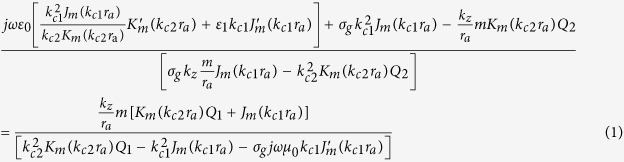


where 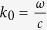, 
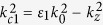
, 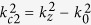, *k*_*z*_ is the wave vector, 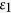 is the permittivity of dielectric medium, *ε*_0_ and *μ*_0_ are the permittivity and permeability of vacuum. *J*_*m*_*(x)*, *K*_*m*_*(x)* are Bessel and modified Bessel functions, *m* (integer) denotes the azimuthal variation number of the field. The other parameters in equation (1) are given in Appendix I of the Supplementary materials.

In the THz regime, only the intraband conductivity is considered^19^,^35^. 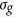 is given as^35^,


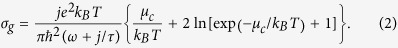


Here 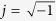, *k*_*B*_ is Boltzmann constant, 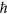 is the reduced Planck constant, *T* is temperature, 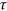 is relaxation time, and 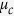 is chemical potential.

### The excitation and transformation of the fundamental SPPs mode

**Figure 2:**
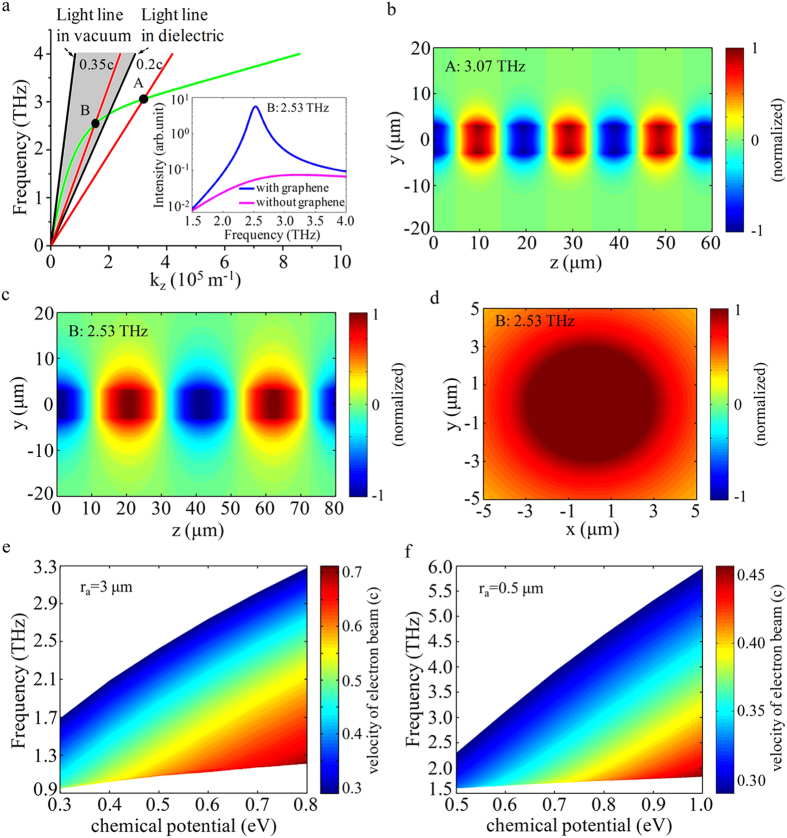
Numerical results of fundamental SPPs mode for the monolayer structure. (**a**) The dispersion curves of fundamental mode (m = 0). The inset is Fourier spectra of radiation intensity from the structure with and without graphene. (**b**) The contour map of electric field *E*_*z*_ in Y-Z plane of excited SPPs at point A. (**c**,**d**) The contour maps of electric field *E*_*z*_ in Y-Z and X-Y planes of excited SPPs at point B. The radiation frequency vs. the chemical potential and beam velocity for the structure with radii r_a_ = 3 μm (**e**) and r_a_ = 0.5 μm (f), respectively.

We now exam the fundamental SPPs TM mode (m = 0). The parameters are *T* = 300*K*, 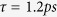
36,37, 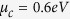, r_a_ = 3μm and 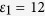. The dispersion curve is shown in Fig. 2(a). The shaded region between the vacuum and dielectric light lines is the Cherenkov radiation zone^38^,^39^. Due to the natural periodicity of 2π of circular cylindrical graphene structure, the SPPs dispersion curve can cross the light line of dielectric entering the Cherenkov radiation zone, making transformation of SPPs into radiation possible. The radiation frequency is determined by the intersection point (the working point) between the beam line and the SPPs dispersion curve. It can be widely tuned with the beam energy.

To make a comparison, we also study the linearly moving electron beam 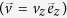 excitation on the circular cylindrical structure. The result shows that only the fundamental SPPs TM mode can be excited. The beam line with velocity 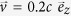 intersects with the dispersion curve at point A outside the radiation zone. The contour map of electric field *E*_*z*_ of excited SPPs at frequency 3.07 THz is shown in Fig. 2(b), indicating that the SPPs cannot be transformed into radiation wave.

Increasing the beam velocity to 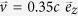, the intersection point B can get inside the radiation zone, now the SPPs can be transformed into radiation. The inset of Fig. 2(a) shows Fourier spectra of radiation power density from the structure with and without graphene, the peak radiation frequency is 2.53 THz. The contour maps of electric field 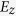 shown in Fig. 2(c,d) indicate that the field distribution of fundamental mode is concentrated in the center of the structure. The peak radiation power density can reach 10^5^ W/cm^2^ for an electron beam with a charge quantity 1pC, and is enhanced more than 300 times over that without graphene SPPs. This radiation power density is two or three orders higher than that of radiation from linearly electron beam excited graphene based grating structure^17^,^18^. The physics origin is that a large fraction of SPPs energy is transformed into radiation in our structure, while in a grating structure only the first negative space harmonic of SPPs with a part energy is transformed into radiation. As discussed in^18^, the performance of radiation from electron beam excited SPPs mainly depends on the quality of graphene, especially its relaxation time. Higher relaxation time leads to stronger and sharper radiation peak, and high performance radiation in the structure can be obtained in a wide relaxation time range based on our calculations given in Appendix IV of the Supplementary materials.

The dependence of the radiation frequency on the chemical potential and beam velocity is shown in Fig. 2(e,f) for the structure with different radii. As the chemical potential increases, the dispersion curve shifts upward due to the increasing surface plasmon frequency. This leads to higher working point and broader dispersion band in the radiation zone, and in turn higher radiation frequency and wider tunable frequency band.

### The excitation and transformation of the hybrid SPPs modes

**Figure 3:**
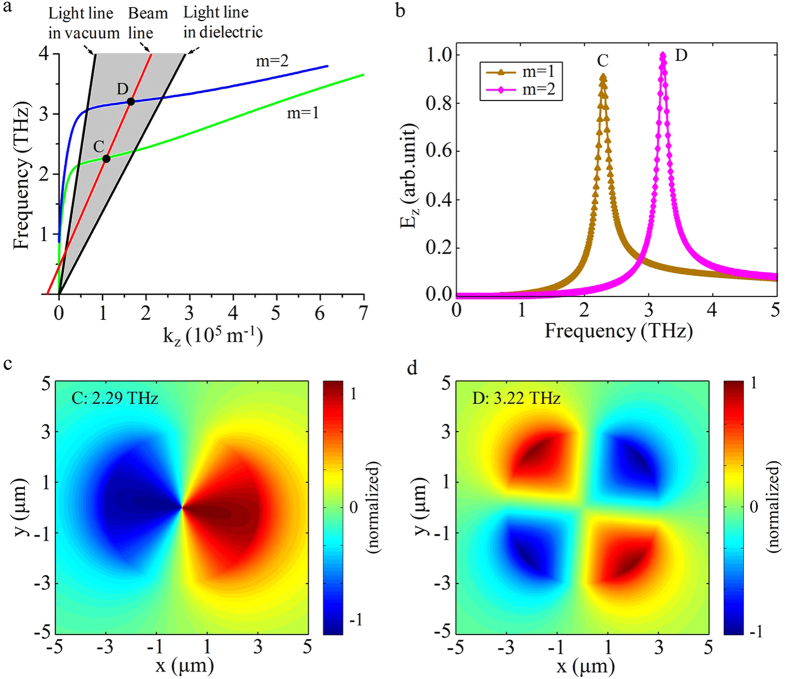
Numerical results of hybrid SPPs modes for the monolayer structure. (**a**) The dispersion curves of hybrid modes (m = 1, 2). (**b**) The Fourier spectra of radiation fields E_z_. (**c**,**d**) The contour maps of electric fields for the first and second hybrid modes.

Figure 3 shows that the hybrid SPPs modes (m ≥ 1), co-existing of TE and TM modes^30^,^32^, can be excited by the CEB in the circular cylindrical structure, and be transformed into Cherenkov radiation in the dielectric medium. The equation of CEB line, 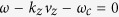, that means the phase-velocity of the excited SPPs are always in synchronization with CEB, and it assures SPPs can gain energy from the electron beam continuously to compensate the loss due to the radiation and decay, where 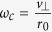 is the cyclotron frequency. The dispersion curves of hybrid modes (m = 1, 2) are shown in Fig. 3(a), the CEB line with velocity 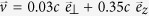 intersects with each mode at points C and D, respectively. Figure 3(b) shows the Fourier spectra of radiation fields E_z_ of first and second hybrid modes, their radiation frequencies are 2.29 THz and 3.22 THz, respectively. The contour maps of electric fields 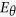 are shown in Fig. 3(c,d).

The circular cylindrical graphene structure can be successfully manufactured^29^. The CEB required in this work can be produced with an adiabatic varying magnetic field section within a uniform longitudinal magnetic field^40^,^41^,^42^. The required magnetic field is only a few Tesla. Here, the energy of the CEB is rather low, the radiation by the CEB directly is neglected^43^.

### The excitation and transformation of SPPs modes in the circular cylindrical double-layer graphene structure

**Figure 4:**
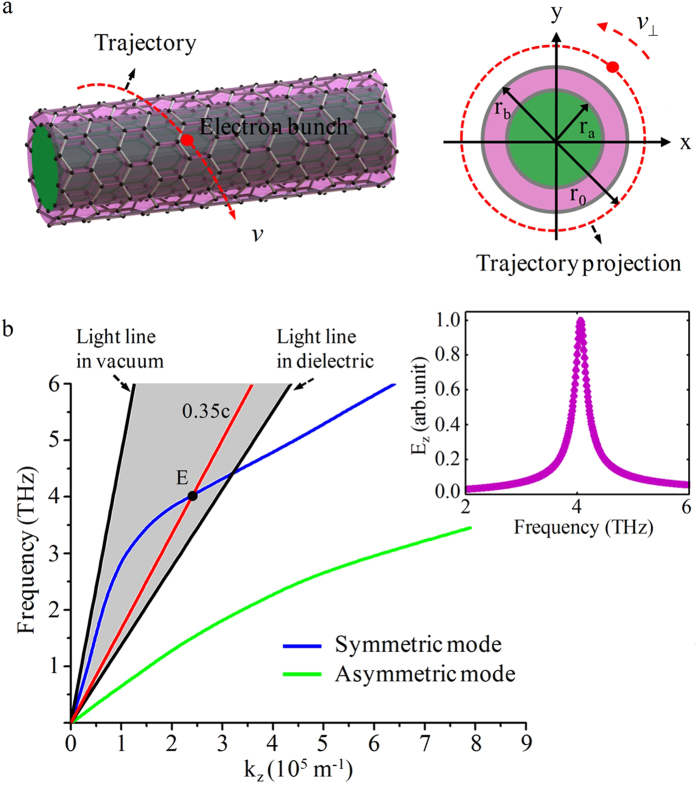
The schematic and dispersion curves of fundamental mode for double-layer structure. (**a**) Schematic of the circular cylindrical double-layer graphene structure with dielectric loading, the radius of the dielectric medium is r_a_, the dielectric film is in the region *r*_*a*_ < *r* < *r*_*b*_, the radius of trajectory projection of CEB is r_0_. (**b**) The dispersion curves of fundamental mode, the parameters are r_a_ = 3 μm, r_b_ = 4 μm, *ε*_1_ = 12, and *ε*_2_ = 2, the inset is the Fourier spectrum of radiation field *E*_*z*_.

The schematic is shown in Fig. 4(a), the graphene layers are separated by a thin dielectric medium 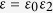 with thickness *r*_b_−*r*_*a*_. The mathematical manipulations can be found in Appendix III of the Supplementary materials. The dispersion curves of fundamental mode (m = 0) are shown in Fig. 4(b). There are two branches of dispersion curves. The upper curve is the symmetric plasmon mode and the lower curve is the asymmetric plasmon mode^44^,^45^,^46^. Only the dispersion curve of symmetric plasmon mode enters the radiation zone and can be transformed into electromagnetic radiation. For a beam line with velocity 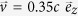, the working point is point E as shown in Fig. 4(b). The spectrum of radiation field *E*_*z*_ is shown in the inset of Fig. 4(b), and the radiation frequency is 4.05 THz.

**Figure 5:**
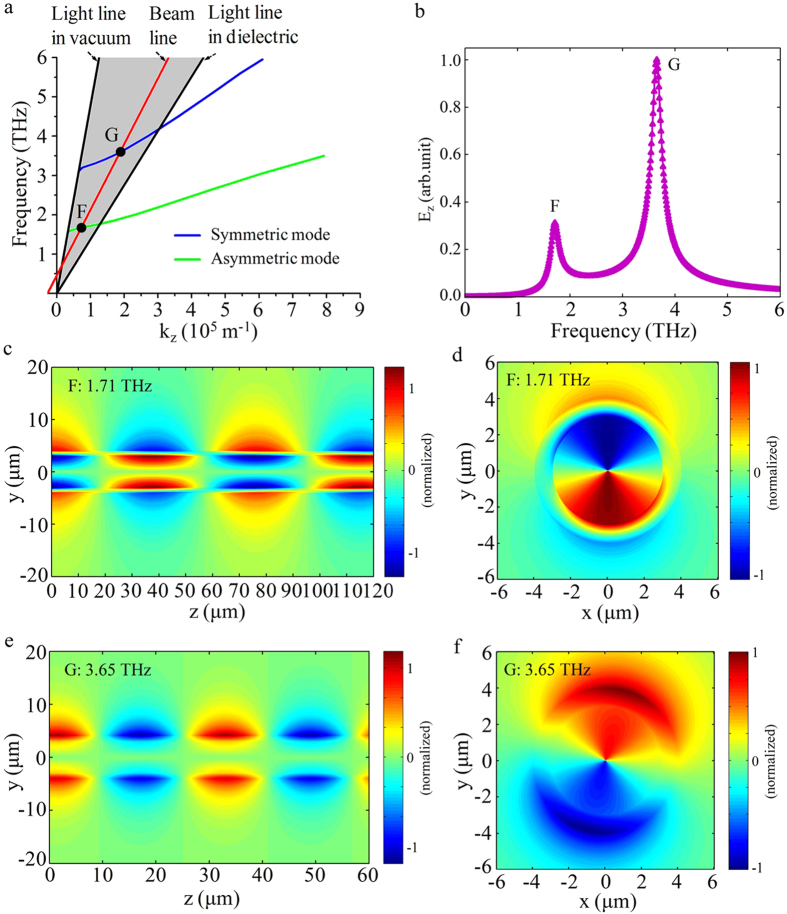
Numerical results of hybrid SPPs modes for the double-layer structure. (**a**) The dispersion curves of first hybrid mode. (**b**) The Fourier spectrum of radiation field E_z_. (**c**,**d**) The contour maps of electric fields E_z_ and *E*_*θ*_ for the asymmetrical plasmon mode. (**e**–**f**) The contour maps of electric fields E_z_ and *E*_*θ*_ for the symmetrical plasmon mode.

A unique and interesting feature of the double-layer structure is that two-color THz radiation can be generated simultaneously from the hybrid SPPs modes. The dispersion curves of hybrid mode (m = 1) are shown in Fig. 5(a). A CEB line with velocity 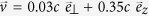 can intersect with the asymmetric and symmetric plasmon modes at point F and G in the radiation zone, respectively. The spectra of the electric fields are shown in the Fig. 5(b), there are two radiation frequencies, 1.71 THz and 3.65 THz, corresponding to the working points F and G. The contour maps of electric fields *E*_*z*_ and 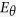 for the two modes are shown in Fig. 5(c–f).

In summary, by making use of the dual natural periodicity of 2π of both the circular cylindrical graphene structure and the CEB, the physical mechanisms of SPPs excitation and transformation into enhanced tunable and coherent THz radiation are presented and investigated. The results show that the radiation power density is enhanced more than 300 times and can reach 10^5^ W/cm^2^ or even higher. The radiation frequency can be tuned in a wide frequency band by adjusting beam energy or the Fermi level of graphene layer. For the double-layer structure, two-color THz radiation can be generated simultaneously from hybrid modes. Therefore, the findings presented here open a promising way for developing room temperature, tunable, coherent and intense THz radiation sources to cover the whole THz band.

## Methods

### Electromagnetic fields produced by CEB

The vector and scalar potentials with Lorentz gauge are utilized to derive the electromagnetic fields produced by the CEB. Solving the wave equations, these two potentials can be obtained, then the electromagnetic fields are obtained.

### Dispersion and output power density

The electromagnetic fields in the circular cylindrical graphene structure can also be achieved by solving the wave equations. Matching the boundary conditions, the dispersion equations and coefficients of fields are obtained, and the output power density can be calculated by integrating the Poynting vector in a unit cell.

## Additional Information

**How to cite this article**: Zhao, T. *et al.* Coherent and Tunable Terahertz Radiation from Graphene Surface Plasmon Polarirons Excited by Cyclotron Electron Beam. *Sci. Rep.* **5**, 16059; doi: 10.1038/srep16059 (2015).

## Supplementary Material

Supplementary Information
