# Supplementary material for: Coherent and Tunable Terahertz Radiation from Graphene Surface Plasmon Polarirons Excited by Cyclotron Electron Beam
Source: Sci Rep. 2015 Nov 3;5:16059. doi: 10.1038/srep16059 (PMC4630615; doi:10.1038/srep16059)
Supplement: Supplementary Information [file srep16059-s1.pdf]

Supplementary material for " Coherent and Tunable Terahertz Radiation from Graphene Surface Plasmon Polarirons Excited by Cyclotron Electron Beam "

Tao Zhao[1, 2], Sen Gong[1, 2], Min Hu[1, 2], Renbin Zhong[1, 2],Diwei Liu[1 ,2],Xiaoxing Chen[1, 2], Ping zhang[1, 2], Xinran Wang[2, 3], Chao Zhang[2, 4], Peiheng Wu[2, 3], Shenggang Liu[* 1, 2]

1. Terahertz Research Center, School of Physical Electronics, University of Electronic Science and Technology of China, Chengdu, Sichuan, 610054, China,

2. Cooperative Innovation Centre of Terahertz Science, Chengdu, Sichuan, 610054, China

3. School of Electronic Science and Engineering, Nanjing University, Nanjing, Jiangsu, 210000, China

4. School of Physics and Institute for Superconducting and Electronic Materials, University of Wollongong, New South Wales 2522, Australia

*liusg@uestc.edu.cn

Appendix I: The electromagnetic fields produced by the cyclotron electron beam

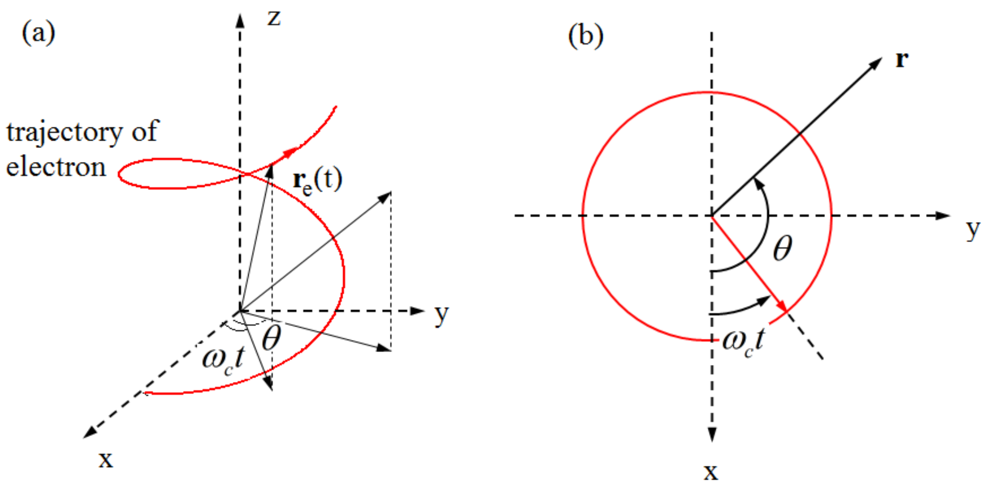

Fig. 1. 1 The schematic of cyclotron electron beam, the electron beam moves at a velocity $\vec{u} = v_\perp \vec{e}_\perp + v_z \vec{e}_z$ with a cyclotron trajectory.

The schematic of an cyclotron electron beam (CEB) is shown in Fig.1.1, the electron beam moves at a velocity $\vec{v} = v_\perp \vec{e}_\perp + v_z \vec{e}_z$ with a cyclotron trajectory. The vector and scalar potentials $\vec{A}$ and φ are used to derive the electromagnetic fields produced by the CEB. The vector and scalar potentials obey the following d'Alembert equations, given below in MKS unit,

$$\nabla^2 \vec{A} - \mu_0 \varepsilon_0 \frac{\partial^2 \vec{A}}{\partial t^2} = -\mu_0 \vec{J} \tag{I.1}$$

$$\nabla^2 \varphi - \mu_0 \varepsilon_0 \frac{\partial^2 \varphi}{\partial t^2} = -\frac{\rho}{\varepsilon_0} \tag{I.2}$$

With Lorentz gauge:

$$\nabla \cdot \vec{A} + \mu_0 \varepsilon_0 \frac{\partial \varphi}{\partial t} = 0 \tag{I.3}$$

Then, the electromagnetic fields $\vec{E}$ and $\vec{H}$ can be obtained:

$$\vec{E} = -\frac{\partial \vec{A}}{\partial t} - \nabla \varphi \qquad \vec{H} = \frac{\nabla \times \vec{A}}{\mu} \tag{I.4}$$

The position and velocity vectors of the CEB can be described in Cartesian co ordinate system.

$$\vec{r}_e(t) = \vec{e}_x x'(t) + \vec{e}_y y'(t) + \vec{e}_z z'(t) \tag{I.5}$$

$$\vec{v}(t) = \vec{e}_\perp v_\perp(t) + \vec{e}_z v_z(t) \tag{I.6}$$

Then, the charge and current density can be expressed.

$$\rho = q\delta\left(\vec{r} - \vec{r}_e(t)\right) = q\delta\left(x - x'(t)\right)\delta\left(y - y'(t)\right)\delta\left(z - z'(t)\right) \tag{I.7}$$

$$\vec{J} = \vec{v}(t)\rho = \left(\vec{e}_\perp v_\perp(t) + \vec{e}_z v_z(t)\right)q\delta\left(x - x'(t)\right)\delta\left(y - y'(t)\right)\delta\left(z - z'(t)\right) \tag{I.8}$$

Where q is the charge quantity, v_z is the z component of velocity, $v_\perp$ is the cyclotron velocity of the beam around z axis. In the cylindrical coordinate syst em,

$$x'(t) = r_0 \cos(\omega_c t) \tag{I.9}$$

$$y'(t) = r_0 \sin(\omega_c t) \tag{I.10}$$

$$z'(t) = v_z t \tag{I.11}$$

Where r_0 is the radius of the trajectory projection of cyclotron electron beam in X-Y plane, $\omega_c = \frac{v_\perp}{r_0}$.

Similarly, the observed position of fields can be expressed in the cylindrical coordinate system.

$$x = r\cos(\theta) \tag{I.12}$$

$$y = r\sin(\theta) \tag{I.13}$$

$$z = z \tag{I.14}$$

Now the charge and current density can be expressed.

$$\rho(\vec{r},t) = q\delta\left(\vec{r} - \vec{r}_e(t)\right) = q \cdot \begin{cases} \delta\left(r\cos(\theta) - r_0\cos(\omega_c t)\right) \\ \cdot\delta\left(r\sin(\theta) - r_0\sin(\omega_c t)\right) \\ \cdot\delta\left(z - v_z t\right) \end{cases} \tag{I.15}$$

$$\vec{J}(\vec{r},t) = \vec{v}(t)\rho = \left(\vec{e}_\perp v_\perp + \vec{e}_z v_z\right)q \cdot \begin{cases} \delta\left(r\cos(\theta) - r_0\cos(\omega_c t)\right) \\ \delta\left(r\sin(\theta) - r_0\sin(\omega_c t)\right) \\ \delta\left(z - v_z t\right) \end{cases} \tag{I.16}$$

The field can be obtained by using the Fourier transform, assume wave factor is in the form $e^{j\vec{k}\cdot\vec{r} - j\omega t}$.

$$\vec{A}(\vec{r},t) = \left(\frac{1}{2\pi}\right)^3 \int \vec{A}\left(\vec{k}\right) e^{j\vec{k}\cdot\vec{r}} e^{-j\omega t} d\vec{k} \tag{I.17}$$

$$\vec{J}(\vec{r},t) = \left(\frac{1}{2\pi}\right)^3 \int \vec{J}\left(\vec{k}\right) e^{j\vec{k}\cdot\left(\vec{r} - \vec{r}_e(t)\right)} d\vec{k} \tag{I.18}$$

Submitting Eq. (I.17) and (I.18) into Eq. (I.1) and (I.2), the vector $\vec{A}$ can be obtained by Fourier transformation in the wave vector space.

$$\vec{A}(\vec{r},t)=\left(\frac{1}{2\pi}\right)^3\int\frac{-\mu_0 q(\vec{e}_\perp v_\perp+\vec{e}_z v_z)e^{j\vec{k}\cdot(\vec{r}-\vec{r}_e(t))}}{(-k_\perp{}^2-k_z{}^2+k_0{}^2)}d\vec{k} \tag{I.19}$$

Based on Lorentz gauge, we can get the scalar potential φ.

$$\varphi=\frac{\nabla\cdot\vec{A}}{j\omega\varepsilon_0\mu_0} \tag{I.20}$$

Based on the Eq. (I.4), we obtain the components of the electric field.

Appendix II: The theory of circular cylindrical monolayer graphene structure

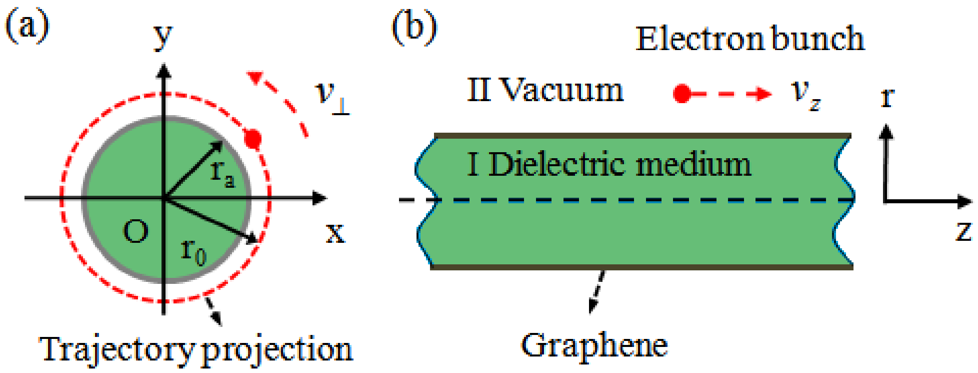

Fig. 2. 1 Schematic of the circular cylindrical monolayer graphene structure with dielectric loading ($\varepsilon=\varepsilon_0\varepsilon_1$), the radius of the dielectric medium is r_a, the radius of the trajectory projection of cyclotron electron beam in X-Y plane is r_0, it moves at a velocity $\vec{v}=v_\perp\vec{e}_\perp+v_z\vec{e}_z$ above the graphene layer with a cyclotron trajectory.

2.1 Dispersion equation

As shown in Fig. 2. 1, the scheme can be divided into two regions. Without charge sources, solving Eq. (I.1) and (I.2) together with the boundary conditions, the fields in region I and II can be obtained. The factor $e^{jk_z z+jm\theta-j\omega t}$ is omitted.

In the region I ($r<r_a$):

$$E_z^I = A_1 k_{c1}^2 J_m(k_{c1}r) \qquad H_z^I = A_2 k_{c1}^2 J_m(k_{c1}r)$$

$$E_r^I = jA_1 k_z k_{c1} J_m{}'(k_{c1}r) - A_2\omega\mu_0 \frac{m}{r} J_m(k_{c1}r)$$

$$E_\theta^I = -A_1 k_z \frac{m}{r} J_m(k_{c1}r) - jA_2\omega\mu_0 k_{c1} J_m{}'(k_{c1}r) \tag{II.1}$$

$$H_r^I = A_1\omega\varepsilon_0\varepsilon_1 \frac{m}{r} J_m(k_{c1}r) + jA_2 k_z k_{c1} J_m{}'(k_{c1}r)$$

$$H_\theta^I = A_1 j\omega\varepsilon_0\varepsilon_1 k_{c1} J_m{}'(k_{c1}r) - A_2 k_z \frac{m}{r} J_m(k_{c1}r)$$

where： $k_{c1}^2 = \varepsilon_1 k_0^2 - k_z^2$ ， $J_m{}'(k_{c1}r) = \frac{1}{2}[J_{m-1}(k_{c1}r) - J_{m+1}(k_{c1}r)]$, $J_0{}'(k_{c1}r) = -J_1(k_{c1}r)$

In the region II ($r \ge r_a$):

$$E_z^{II} = A_3 k_{c2}^2 K_m(k_{c2}r), \quad H_z^{II} = A_4 k_{c2}^2 K_m(k_{c2}r)$$

$$E_r^{II} = -jk_z k_{c2} A_3 K_m{}'(k_{c2}r) + \omega\mu_0 \frac{m}{r} A_4 K_m(k_{c2}r)$$

$$E_\theta^{II} = k_z \frac{\mathrm{m}}{r} A_3 K_m(k_{c2}r) + j\omega\mu_0 k_{c2} A_4 K_m'(k_{c2}r) \tag{II.2}$$

$$H_r^{II} = -jk_z k_{c2} A_4 K_m'(k_{c2}r) - \frac{\omega\varepsilon_0}{r} mA_3 K_m(k_{c2}r)$$

$$H_\theta^{II} = \frac{k_z}{r} mA_4 K_m(k_{c2}r) - j\omega\varepsilon_0 k_{c2} A_3 K_m'(k_{c2}r)$$

where：

$$k_{c2}^2 = k_z^2 - k_0^2 \,, \qquad K_m{}'(k_{c2}r) = -\frac{1}{2}[K_{m-1}(k_{c2}r) + K_{m+1}(k_{c2}r)],\ K_0{}'(k_{c2}r) = -K_1(k_{c2}r)$$

Assuming that monolayer graphene is atomically thin, it can be regarded as a conductive surface with conductivity σ_g. The boundary conditions are shown as below:

$$E_z^I\Big|_{r=r_a} = E_z^{II}\Big|_{r=r_a}, \qquad E_\theta^I\Big|_{r=r_a} = E_\theta^{II}\Big|_{r=r_a}$$

$$(H_z^I - H_z^{II})\Big|_{r=r_a} = \sigma_g E_\theta^I\Big|_{r=r_a}, \quad (H_\theta^{II} - H_\theta^I)\Big|_{r=r_a} = \sigma_g E_z^I\Big|_{r=r_a} \tag{II.3}$$

After substituting Eq. (II.1) and (II.2) into the above boundary conditions, the dispersion equation of circular cylindrical graphene structure with dielectric loading is obtained,

$$\frac{j\omega\varepsilon_0[\frac{k_{c1}^2 J_m(k_{c1}r_a)}{k_{c2}K_m(k_{c2}r_a)}K_m'(k_{c2}r_a)+\varepsilon_1 k_{c1}J_m{}'(k_{c1}r_a)]+\sigma_g k_{c1}^2 J_m(k_{c1}r_a)-\frac{k_z}{r_a}mK_m(k_{c2}r_a)Q_2}{[\sigma_g k_z\frac{m}{r_a}J_m(k_{c1}r_a)-k_{c2}^2K_m(k_{c2}r_a)Q_2]}$$

$$=\frac{\frac{k_z}{r_a}m[K_m(k_{c2}r_a)Q_1+J_m(k_{c1}r_a)]}{[k_{c2}^2K_m(k_{c2}r_a)Q_1-k_{c1}^2J_m(k_{c1}r_a)-\sigma_g j\omega\mu_0 k_{c1}J_m{}'(k_{c1}r_a)]} \tag{II.4}$$

where:

$$Q_1=-\frac{k_{c1}J_m{}'(k_{c1}r_a)}{k_{c2}K_m'(k_{c2}r_a)},\quad Q_2=\frac{jk_z\frac{\mathrm{m}}{r_a}J_m(k_{c1}r_a)(\frac{k_{c1}^2}{k_{c2}^2}+1)}{\omega\mu_0 k_{c2}K_m'(k_{c2}r_a)}$$

2.2 Power density

2.2.1 Excitation of fundamental SPPs mode by a linearly moving electron beam

Because the fundamental SPPs mode (m=0) is a transverse magnetic (TM) mode, it can be excited by the TM evanescent fields produced by a linearly moving electron beam (the particular case of CEB without rotating velocity). The electromagnetic fields produced by the linearly moving electron can be expressed as below [1].

$$E_z^i=-A_0 e^{jk_z z}\begin{cases}J_0(k_{ci}r_0)H_0^{(1)}(k_{ci}r) & (r>r_0)\\ J_0(k_{ci}r)H_0^{(1)}(k_{ci}r_0) & (r<r_0)\end{cases},$$

$$H_\varphi^i=\frac{j\omega\varepsilon_0 A_0}{k_{ci}}e^{jk_z z}\begin{cases}J_0(k_{ci}r_0)H_1^{(1)}(k_{ci}r) & (r>r_0)\\ J_1(k_{ci}r)H_0^{(1)}(k_{ci}r_0) & (r<r_0)\end{cases} \tag{II.5}$$

where $A_0=\frac{\pi\omega\mu_0 q}{2}(1-1/\beta^2),\beta=\frac{v_z}{c}$

For the fundamental mode, the TM fields in the structure can be expressed as below.

In the region I ($r<r_a$):

$$E_z^I=A_1 k_{c1}^2 J_0(k_{c1}r)$$

$$E_r^I=jA_1 k_z k_{c1}J_0{}'(k_{c1}r) \tag{II.6}$$

$$H_\theta^I=A_1 j\omega\varepsilon_0\varepsilon k_{1c}J_1{}'(k_{0c}r)$$

where: $k_{c1}^2=\varepsilon_1 k_0^2-k_z^2$, $J_0{}'(k_{c1}r)=-J_1(k_{c1}r)$

In the region II ($r\ge r_a$):

$$E_z^{II}=A_3 k_{c2}^2 K_0(k_{c2}r)$$

$$E_r^{II}=-jk_z k_{c2}A_3 K_0{}'(k_{c2}r) \tag{II.7}$$

$$H_\theta^{II}=-j\omega\varepsilon_0 k_{c2}A_3 K_0'(k_{c2}r)$$

where：

$$k_{c2}^2 = k_z^2 - k_0^2, \quad K_0'(k_{c2}r) = -K_1(k_{c2}r)$$

The boundary conditions can be written as

$$E_z^I\Big|_{r=r_a} = (E_z^{II} + E_z^i)\Big|_{r=r_a}, (H_\theta^{II} + H_\varphi^i - H_\varphi^I)\Big|_{r=r_a} = \sigma_g E_z^I\Big|_{r=r_a} \qquad \text{(II.8)}$$

By solving the above boundary conditions, the fields coefficients can be determined.

2.2.2 Excitation of hybrid modes by CEB

The electromagnetic fields in the structure and produced by CEB are obtained in the previous paragraphs. The boundary conditions can be written as,

$$\begin{aligned} &E_z^I\Big|_{r=r_a} = (E_z^{II} + E_z^i)\Big|_{r=r_a}, && E_\theta^I\Big|_{r=r_a} = (E_\theta^{II} + E_\theta^i)\Big|_{r=r_a}, \\ &(H_z^I - H_z^{II} - H_z^i)\Big|_{r=r_a} = \sigma_g E_\theta^I\Big|_{r=r_a}, && (H_\theta^{II} + H_\theta^i - H_\theta^I)\Big|_{r=r_a} = \sigma_g E_z^I\Big|_{r=r_a} \end{aligned} \qquad \text{(II.9)}$$

Submitting the electromagnetic fields into the above boundary conditions, the field coefficients can be obtained.

The excited SPPs are transformed into radiation in the dielectric medium when the Cherenkov radiation condition is satisfied. The radiation power density can be calculated by the following equation,

$$P_z = -\frac{1}{2}\text{Re}[\int\int E_r^I \times H_\theta^{I*} r dr d\theta] \qquad \text{(II.10)}$$

Appendix III: The theory of circular cylindrical double-layer graphene structure

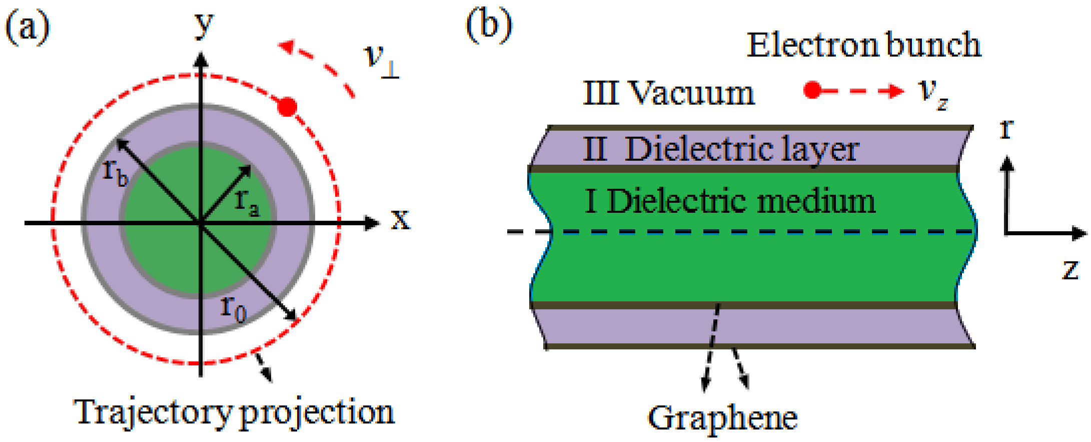

Fig. 3. 1 Schematic of circular cylindrical double-layer graphene structure with dielectric loading, the radius of the dielectric medium is r_a, the dielectric film is in the region $r_a < r < r_b$, and the radius of the trajectory projection of electron beam is r_0.

3.1 Dispersion equation

As shown in Fig. 3.1, the scheme can be divided into three regions. Without charge sources, solving Eq. (I.1) and (I.2) together with the boundary conditions, the fields in region I, II and III can be obtained. The factor $e^{jk_z z+jm\theta-j\omega t}$ is omitted.

In the region I ($r<r_a$):

$$E_z^I = A_1 k_{c1}^2 J_m(k_{c1}r) \qquad H_z^I = A_2 k_{c1}^2 J_m(k_{c1}r)$$

$$E_r^I = jA_1 k_z k_{c1} J_m{}'(k_{c1}r) - A_2\omega\mu_0 \frac{m}{r} J_m(k_{c1}r)$$

$$E_\theta^I = -A_1 k_z \frac{m}{r} J_m(k_{c1}r) - jA_2\omega\mu_0 k_{c1} J_m{}'(k_{c1}r) \qquad \text{(III.1)}$$

$$H_r^I = A_1\omega\varepsilon_0\varepsilon_1 \frac{m}{r} J_m(k_{c1}r) + jA_2 k_z k_{c1} J_m{}'(k_{c1}r)$$

$$H_\theta^I = A_1 j\omega\varepsilon_0\varepsilon_1 k_{c1} J_m{}'(k_{c1}r) - A_2 k_z \frac{m}{r} J_m(k_{c1}r)$$

where: $k_{c1}^2=\varepsilon_1 k_0^2 - k_z^2$, $J_m{}'(k_{c1}r)=\frac{1}{2}[J_{m-1}(k_{c1}r)-J_{m+1}(k_{c1}r)]$, $J_0{}'(k_{c1}r)=-J_1(k_{c1}r)$

In the region II ($r_a<r<r_b$):

$$E_z^{II} = A_3 k_{c2}^2 I_m(k_{c2}r) + A_4 k_{c2}^2 K_m(k_{c2}r), \quad H_z^{II} = A_5 k_{c2}^2 I_m(k_{c2}r) + A_6 k_{c2}^2 K_m(k_{c2}r)$$

$$E_r^{II} = -jk_z k_{c2}[A_3 I_m'(k_{c2}r) + A_4 K_m{}'(k_{c2}r)] + \omega\mu_0 \frac{m}{r}[A_5 I_m(k_{c2}r) + A_6 K_m(k_{c2}r)]$$

$$E_\theta^{II} = k_z \frac{\mathrm{m}}{r}[A_3 I_m(k_{c2}r) + A_4 K_m(k_{c2}r)] + j\omega\mu_0 k_{c2}[A_5 I_m'(k_{c2}r) + A_6 K_m'(k_{c2}r)] \qquad \text{(III.2)}$$

$$H_r^{II} = -jk_z k_{c2}[A_5 I_m'(k_{c2}r) + A_6 K_m'(k_{c2}r)] - \frac{\omega\varepsilon_0\varepsilon_2}{r} m[A_3 I_m(k_{c2}r) + A_4 K_m(k_{c2}r)]$$

$$H_\theta^{II} = \frac{k_z}{r} m[A_5 I_m(k_{c2}r) + A_6 K_m(k_{c2}r)] - j\omega\varepsilon_0\varepsilon_2 k_{c2}[A_3 I_m'(k_{c2}r) + A_4 K_m'(k_{c2}r)]$$

where:

$k_{c2}^2 = k_z^2 - \varepsilon_2 k_0^2$, $I_m{}'(k_{c2}r)=\frac{1}{2}[I_{m-1}(k_{c2}r)+I_{m+1}(k_{c2}r)]$, $I_0{}'(k_{c2}r)=I_1(k_{c2}r)$,

$K_m{}'(k_{c2}r) = -\frac{1}{2}[K_{m-1}(k_{c2}r)+K_{m+1}(k_{c2}r)]$, $K_0{}'(k_{c2}r)=-K_1(k_{c2}r)$

In the region III ($r\ge r_b$):

$$E_z^{III} = A_7 k_{c3}^2 K_m(k_{c3}r), \qquad H_z^{III} = A_8 k_{c3}^2 K_m(k_{c3}r)$$
$$E_r^{III} = -jk_z k_{c3} A_7 K_m{}'(k_{c3}r) + \omega\mu_0 \frac{m}{r} A_8 K_m(k_{c3}r)$$
$$E_\theta^{III} = k_z \frac{\mathrm{m}}{r} A_7 K_m(k_{c3}r) + j\omega\mu_0 k_{c3} A_8 K_m'(k_{c3}r) \tag{III.3}$$
$$H_r^{III} = -jk_z k_{c3} A_8 K_m'(k_{c3}r) - \frac{\omega\varepsilon_0}{r} m A_7 K_m(k_{c3}r)$$
$$H_\theta^{III} = \frac{k_z}{r} m A_8 K_m(k_{c3}r) - j\omega\varepsilon_0 k_{c3} A_7 K_m'(k_{c3}r)$$

where：

$$k_{c3}^2 = k_z^2 - k_0^2\,, \quad K_m{}'(k_{c3}r) = -\frac{1}{2}[K_{m-1}(k_{c3}r) + K_{m+1}(k_{c3}r)],\ K_0{}'(k_{c3}r) = -K_1(k_{c3}r)$$

The boundary conditions can be written as,

$$E_z^I\Big|_{r=r_a} = E_z^{II}\Big|_{r=r_a}, \qquad E_\theta^I\Big|_{r=r_a} = E_\theta^{II}\Big|_{r=r_a},$$
$$(H_z^I - H_z^{II})\Big|_{r=r_a} = \sigma_g E_\theta^I\Big|_{r=r_a}, \quad (H_\theta^{II} - H_\theta^I)\Big|_{r=r_a} = \sigma_g E_z^I\Big|_{r=r_a} \tag{III.4}$$

$$E_z^{II}\Big|_{r=r_b} = E_z^{III}\Big|_{r=r_b}, \qquad E_\theta^{II}\Big|_{r=r_b} = E_\theta^{III}\Big|_{r=r_b},$$
$$(H_z^{II} - H_z^{III})\Big|_{r=r_b} = \sigma_g E_\theta^{III}\Big|_{r=r_b}, \quad (H_\theta^{III} - H_\theta^{II})\Big|_{r=r_b} = \sigma_g E_z^{III}\Big|_{r=r_b} \tag{III.5}$$

Submitting the electromagnetic fields into the above boundary conditions, the dispersion equation can be obtained.

3.2 Power density

3.2.1 Excitation of fundamental mode by a linearly moving electron beam

For the fundamental mode (m=0), the electromagnetic fields in the structure can be expressed as,

In the region I ($r < r_a$):

$$E_z^I = A_1 k_{c1}^2 J_0(k_{c1}r)$$
$$E_r^I = jA_1 k_z k_{c1} J_0{}'(k_{c1}r) \tag{III.6}$$
$$H_\theta^I = A_1 j\omega\varepsilon_0\varepsilon_1 k_{1c} J_1{}'(k_{0c}r)$$

where： $k_{c1}^2 = \varepsilon_1 k_0^2 - k_z^2\,, \quad J_0{}'(k_{c1}r) = -J_1(k_{c1}r)$

In the region II ($r_a < r < r_b$):

$$
\begin{aligned}
E_z^{II} &= A_3 k_{c2}^2 I_0(k_{c2}r) + A_4 k_{c2}^2 K_0(k_{c2}r) \\
E_r^{II} &= -jk_z k_{c2}[A_3 I_0'(k_{c2}r) + A_4 K_0{}'(k_{c2}r)] \\
H_\theta^{II} &= -j\omega\varepsilon_0\varepsilon_2 k_{c2}[A_3 I_0'(k_{c2}r) + A_4 K_0'(k_{c2}r)]
\end{aligned}
\qquad \text{(III.7)}
$$

where：

$k_{c2}^2 = k_z^2 - \varepsilon_2 k_0^2$， $I_0{}'(k_{c2}r) = I_1(k_{c2}r)$， $K_0{}'(k_{c2}r) = -K_1(k_{c2}r)$

In the region III ($r \geq r_b$):

$$
\begin{aligned}
E_z^{III} &= A_7 k_{c3}^2 K_0(k_{c3}r) \\
E_r^{III} &= -jk_z k_{c3} A_7 K_0{}'(k_{c3}r) \\
H_\theta^{III} &= -j\omega\varepsilon_0 k_{c3} A_7 K_0'(k_{c3}r)
\end{aligned}
\qquad \text{(III.8)}
$$

with $k_{c3}^2 = k_z^2 - k_0^2$

The boundary conditions can be written as,

$$
\begin{aligned}
&E_z^I\big|_{r=r_a} = E_z^{II}\big|_{r=r_a}, \qquad (H_\theta^{II} - H_\theta^I)\big|_{r=r_a} = \sigma_g E_z^I\big|_{r=r_a} \\
&E_z^{II}\big|_{r=r_b} = (E_z^{III} + E_z^i)\big|_{r=r_b}, \qquad (H_\theta^{III} + H_\theta^i - H_\theta^{II})\big|_{r=r_b} = \sigma_g E_z^{II}\big|_{r=r_b}
\end{aligned}
\qquad \text{(III.9)}
$$

By submitting the electromagnetic fields into the above boundary conditions, the fields coefficients can be obtained.

2.2.2 Excitation of hybrid modes by CEB

The hybrid modes can be excited by the CEB, the boundary conditions can be written as,

$$
\begin{aligned}
&E_z^I\big|_{r=r_a} = E_z^{II}\big|_{r=r_a}, \qquad E_\theta^I\big|_{r=r_a} = E_\theta^{II}\big|_{r=r_a} \\
&(H_z^I - H_z^{II})\big|_{r=r_a} = \sigma_g E_\theta^I\big|_{r=r_a}, \quad (H_\theta^{II} - H_\theta^I)\big|_{r=r_a} = \sigma_g E_z^I\big|_{r=r_a}
\end{aligned}
\qquad \text{(III.10)}
$$

$$
\begin{aligned}
&E_z^{II}\big|_{r=r_b} = E_z^{III} + E_z^i\big|_{r=r_b}, \qquad E_\theta^{II}\big|_{r=r_b} = (E_\theta^{III} + E_\theta^i)\big|_{r=r_b} \\
&(H_z^{II} - H_z^{III} - H_z^i)\big|_{r=r_b} = \sigma_{g2} E_\theta^{III}\big|_{r=r_b}, \ (H_\theta^{III} + H_\theta^i - H_\theta^{II})\big|_{r=r_b} = \sigma_{g2} E_z^{III}\big|_{r=r_b}
\end{aligned}
\qquad \text{(III.11)}
$$

Submitting the electromagnetic fields into the above boundary conditions, the fields coefficients can be obtained.

Appendix IV: Influence of relaxation time of graphene on radiation performance

The performance of radiation from electron beam excited SPPs is mainly dependent on the quality of graphene, especially its relaxation time τ. In this section, we

discuss the influence of τ on the radiation performance. In the manuscript, the value of $\tau = 1.2\,ps$ is used based on recent high-quality graphene [2]. While, it is known that the most CVD graphene is with low-quality, and its relaxation time would further decrease when placed on a dielectric substrate due to the extrinsic scattering [3], which leads to the small relaxation time of the order of 0.1 *ps* [4]. However, recent advances in CVD fabrication of high-quality graphene make long relaxation time available [5]. Fig. 4. 1 shows the results of normalized attenuation constants of graphene SPPs and Fourier spectra of radiation intensity as a function of frequency for relaxation time $\tau = 1.2\,ps$ and $\tau = 0.1\,ps$, respectively. And other parameters are the same as those used in Fig. 2 in the manuscript. The attenuation constant of relaxation time $\tau = 0.1\,ps$ is much larger than that of $\tau = 1.2\,ps$, especially in the long-wavelength regime where the radiation intensity is very weak.

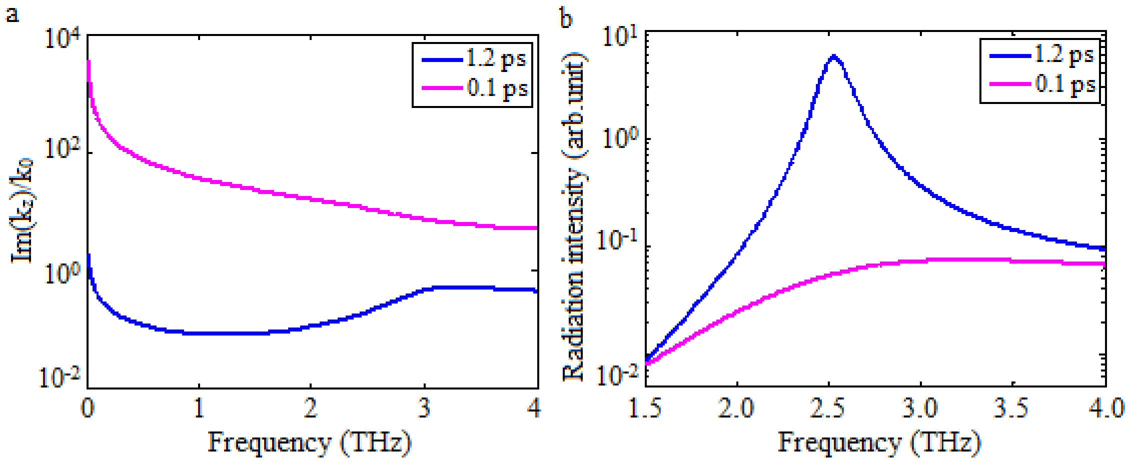

Fig. 4. 1 (a) Normalized attenuation constants of graphene SPPs and (b) Fourier spectra of radiation intensity as a function of frequency for relaxation time $\tau = 1.2\,ps$ and $\tau = 0.1\,ps$.

However, the situation is much better when the relaxation time increases to 0.2 *ps*, as shown in Fig. 4. 2. The ratio of attenuation constants for relaxation time $\tau = 0.2\,ps$ and $\tau = 1.2\,ps$ is less than 10, so the radiation intensity is stronger for $\tau = 0.2\,ps$

than that for $\tau = 0.1ps$. But the radiation intensity at the radiation peak is only thirtieth of that for $\tau = 1.2ps$. Fig. 4. 3 shows Fourier spectra of radiation intensity for different relaxation times. Higher relaxation time leads to stronger and sharper radiation peak, which has been analyzed by [6]. This is caused by stronger resonant strength and lower resonant damping. While the radiation intensity for $\tau = 1.2ps$ is several times higher than those for $\tau \geq 0.4ps$. So high performance radiation can be obtained in a wide relaxation time range.

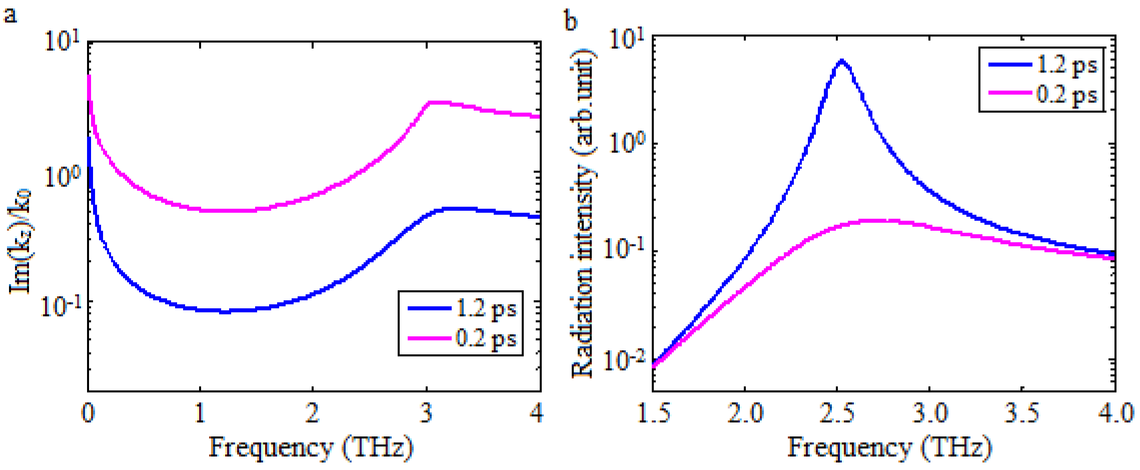

Fig. 4. 2 (a) Normalized attenuation constants of graphene SPPs and (b) Fourier spectra of radiation intensity as a function of frequency for relaxation time $\tau = 1.2ps$ and $\tau = 0.2ps$.

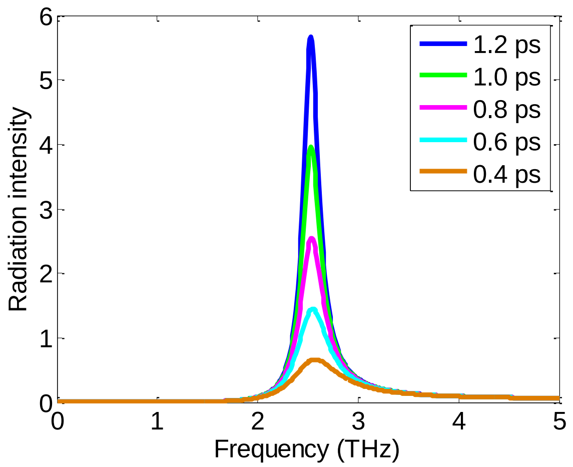

Fig. 4. 3 Fourier spectra of radiation intensity as a function of frequency for different relaxation times.

Reference:

1. Liu, S. *et. el.* Phys. Rev. Lett. **109**, 153902 (2012).

2. Dean, C. *et al.* Boron nitride substrates for high-quality graphene electronics.

Nature Nanotechnol. **5**, 722-726 (2010).
3. Chen, J., Jang, C., Xiao, S., Ishigami, M. & Fuhrer, M. Nat. Nanotechnol. Intrinsic and extrinsic performance limits of graphene devices on SiO_2 . **3**, 206 (2008).
4. Tassin, P., Koschny, T. & Soukoulis, C. Graphene for Terahertz Applications. Science. **341**, 620 (2013).
5. Hao,Y. *et al*. The role of surface oxygen in the growth of large single-crystal graphene on copper. Science. **342**, 720 (2013).
6. Zhan, T. *et al*. Tunable terahertz radiation from graphene induced by moving electrons. Phys. Rev. B. **89**, 245434 (2014).
